# Advancements in Flexible Sensors for Monitoring Body Movements during Sleep: A Review

**DOI:** 10.3390/s24165091

**Published:** 2024-08-06

**Authors:** Zongyi Jiang, Yee Sum Lee, Yunzhong Wang, Honey John, Liming Fang, Youhong Tang

**Affiliations:** 1 Institute for NanoScale Science and Technology, Medical Device Research Institute, College of Science and Engineering, Flinders University, Adelaide 5042, Australia; jian0393@flinders.edu.au (Z.J.); lee1961@flinders.edu.au (Y.S.L.); steven.wang@flinders.edu.au (Y.W.); honey@cusat.ac.in (H.J.); 2 Inter University Centre for Nanomaterials and Devices, Cochin University of Science and Technology, Kochi 682022, India; 3 National Engineering Research Centre for Tissue Restoration and Reconstruction, South China University of Technology, Guangzhou Higher Education Mega Centre, Panyu District, Guangzhou 510006, China

**Keywords:** sleep monitoring, body movements, flexible sensors, wearable devices, piezoelectric nanogenerator, triboelectric nanogenerator

## Abstract

Sleep plays a role in maintaining our physical well-being. However, sleep-related issues impact millions of people globally. Accurate monitoring of sleep is vital for identifying and addressing these problems. While traditional methods like polysomnography (PSG) are commonly used in settings, they may not fully capture natural sleep patterns at home. Moreover, PSG equipment can disrupt sleep quality. In recent years, there has been growing interest in the use of sensors for sleep monitoring. These lightweight sensors can be easily integrated into textiles or wearable devices using technology. The flexible sensors can be designed for skin contact to offer continuous monitoring without being obtrusive in a home environment. This review presents an overview of the advancements made in flexible sensors for tracking body movements during sleep, which focus on their principles, mechanisms, and strategies for improved flexibility, practical applications, and future trends.

## 1. Introduction

Sleep is a complex physiological activity that is crucial for our mental and physical well-being. When we sleep, our bodies undergo changes such as fluctuations in heart rate, breathing, brain activity, and body movements. These changes come with different stages of sleep, and each stage has distinct restorative and cognitive functions [1]. Sleep problems are quite common worldwide, affecting around 40% of people [2]. These issues can manifest in ways like insomnia, sleep apnea, or restless leg syndrome. They can greatly impact a person’s quality of life by causing fatigue and cognitive impairments and increasing the risk of health conditions [3]. Sleep is not one uniform state, but rather a series of distinct stages with specific behaviors. It involves transitioning between non-rapid eye movement (NREM) sleep and rapid eye movement (REM) sleep [4]. The NREM stage includes the N1, N2, and N3 stages, which progressively become deeper. During REM sleep, rapid eye movements occur beneath the eyelids, and most dreaming happens at this time [5]. Typically, adults go through four to six cycles of these stages each night, with a cycle lasting around ninety minutes. Each stage possesses its characteristics, as outlined in Table 1 [6].

**Figure 1:**
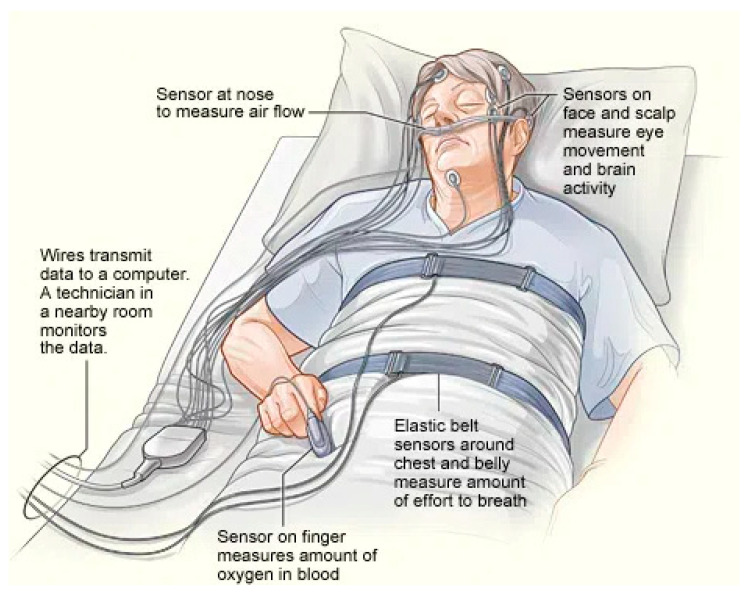
Clinical sleep-monitoring polysomnography, displaying various electrodes connected to different body parts. Source: National Heart Lung and Blood Institute (NIH). The figure was taken from Wikimedia Commons-Sleep Studies-https://commons.wikimedia.org/wiki/File:Sleep_studies.jpg (accessed on 13 July 2024).

Accurate monitoring of sleep plays an important role in the diagnosis and treatment of sleep disorders. Traditional methods like polysomnography (PSG) are widely recognized as the gold standard for assessing sleep. In a PSG setup, the individual spends a night in a sleep lab while being wired with monitoring devices, as shown in Figure 1. There are several limitations, although PSG provides comprehensive sleep data. The controlled environment of a clinic may not fully represent how people sleep at home naturally. Moreover, the equipment used in PSG can be intrusive and uncomfortable, potentially disrupting sleep quality [7]. Actigraphy through devices like smartwatches and fitness trackers presents a user-friendly and non-invasive method for tracking sleep. These devices usually use accelerometers to detect body movements and estimate stages of sleep. Despite offering insights into sleeping habits, these devices still face challenges in terms of accuracy and comprehensiveness [8,9].

**Figure 2:**
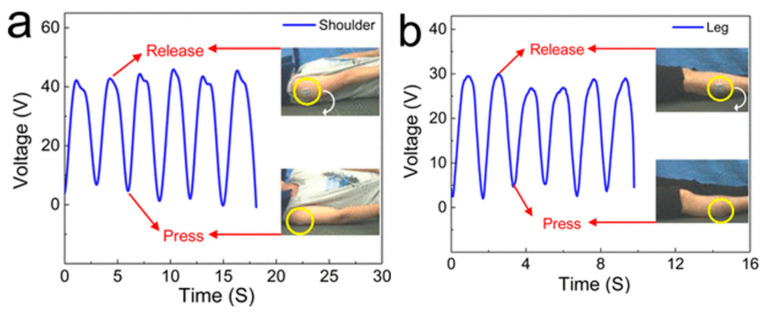
Application of the flexible sensor to monitor body movement while sleeping. Voltage variations over time are recorded from sensors attached to (**a**) a shoulder and (**b**) a leg. The pictures show graphs both before and after the human body overturned [13]. Triboelectric nanogenerator sensors were attached to the shoulder and leg to detect overturning movements during sleep. A noticeable drop in the generated the output of open-circuit voltage indicates that the person has turned to the side, reducing the distance between electrodes. When the person returns to a supine position, pressure is released and the spring leaf rebounds, separating the electrodes. This movement is reflected in the change of the open-circuit voltage output. Thus, the output voltage signal can record and monitor human movement during sleep. Reprinted (adapted) with permission from ACS Nano 2016, 10, 8, 8097–8103. Copyright 2016 American Chemical Society.

**Figure 3:**
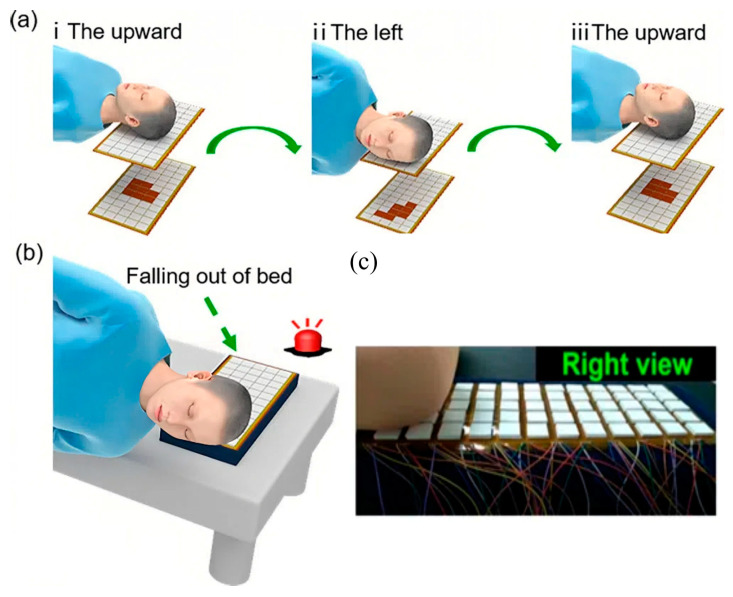
The flexible sensor is used as a smart pillow to track head movements during sleep [16]. (**a**) How the head rotates in different directions. (**b**) A situation where a person could fall off their bed to activate the alarm system once the head approaches the edge column (**c**) A physical image of the smart pillow, which uses a multichannel acquisition system to collect output signals. When the head rests on the smart pillow, the triboelectric nanogenerator units generate signals captured by the acquisition card and processed by a computer program. In experiments, a head model was manually moved on the pillow to simulate sleep movements. It demonstrates real-time head movements from upward to left and back, with voltage changes in each channel; not only can the distribution of the head pressure be clearly reflected, but the movement of the head can also be recorded and detected at the same time. Reprinted (adapted) with permission from ACS Appl. Mater. Interfaces 2022, 14, 20, 23998–24007. Copyright 2022 American Chemical Society.

There are some key body parts that will have sensors attached to collect the data to assess sleep quality and diagnose sleep disorders. Limb movements: Monitoring leg and arm movements is crucial for identifying periodic limb movement disorder (PLMD) and restless leg syndrome (RLS). Electrodes, such as electromyography (EMG), placed on the chin can detect muscle activity associated with limb movements [10]. Chest and abdominal movements: They are monitored using inductance PSG belts fastened around the abdomen and chest. These belts measure changes in chest and abdominal wall circumference, reflecting breathing patterns and potential sleep apnea [11]. Eyes: Electrooculography (EOG) electrodes placed around the eyes detect eye movements. Monitoring REM sleep is crucial for analyzing sleep stages and potential sleep disorders like REM sleep behavior disorder [12]. Based on the detection requirements for sleep monitoring, flexible monitors can conform to the body’s contours, allowing them to capture a wider range of body movements, including subtle shifts and positions. In recent decades, flexible sensors have been used as a positive alternative for detecting body movements in sleep monitoring. As shown in Figure 2, Song et al. used and verified the triboelectric nanogenerator (TENG) as flexible sensor on the shoulder and leg for monitoring body movements during sleep [13]. Recent advancements in sleep monitoring technologies have explored the integration of flexible sensors into everyday textiles. For instance, there are now textiles that use triboelectric nanogenerator arrays, offering a promising solution for self-powered sleep monitoring. These smart textiles can be seamlessly integrated into bedding, allowing for unobtrusive monitoring of sleep at home [14,15]. The flexible sensor can be directly placed onto a shoulder to detect overturning movements during sleep (Figure 2a). A drop in volt output indicates that the sensor is pressed as the person turns toward the side. In a supine posture, pressure release allows the sensor to rebound, separating the electrodes and changing the output of open-circuit voltage from Leg movements can also be monitored with a sensor fixed on the leg (Figure 2b). Postural change is crucial for evaluating sleep quality. Real-time head movements are key indicators reflecting the overall body state during sleep. As shown in Figure 3, Kou et al. developed a smart pillow based on a flexible sensor for monitoring head movements during sleep. It was developed by using porous PDMS and TENG pressure-sensing arrays for mapping pressure distribution and monitoring motion trajectories, particularly head movements and body turnover, during sleep [16]. The flexible sensors are lightweight, conformable, and can be designed for direct skin contact, providing more precise monitoring and making them a potentially more user-friendly option for long-term sleep monitoring at home.

This review explores the latest advancements in sensors used for tracking body movements during sleep. It delves into the principles, mechanisms, and material approaches that improve sensor flexibility, pinpointing areas needing research and predicting trends. The unique and significant aspect of this review is its assessment of both the practical applications of flexible sensor technologies in real-life scenarios and their potential to revolutionize sleep-monitoring practices. By offering insights into sensor materials and innovative strategies for flexibility, this review aims to guide future research and development efforts in the field, ultimately improving the accuracy and user experience of sleep monitoring.

## 2. Working Mechanisms of Flexible Sensors

**Figure 4:**
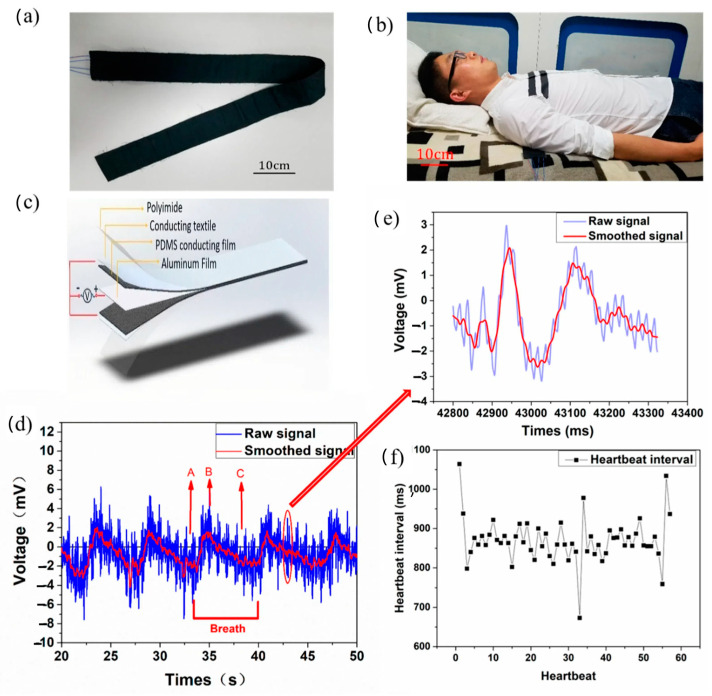
The sleep monitoring belt uses TENG to track breath and heartbeat signals. (**a**) Sleep monitoring belt. (**b**) User sleeping with the belt. (**c**) TENG belt structure. (**d**) Real-time breath and heartbeat voltage. The smoothed signal revealed our tester’s breath pattern after data processing, showing a typical slow wave shape indicative of the breathing signal. Our tester completed about five breathing cycles in a prone position over 30 s. The breathing signals showed two distinct stages: point A to point B represents inhalation and point B to point C represents exhalation. Inhalation is shorter than exhalation—the former takes approximately 2 s, and the latter approximately 5 s. (**e**) Enlarged heartbeat signal. (**f**) Heartbeat interval distribution. Reused and modified with permissions after [22].

Piezoresistive sensors, piezoelectric nanogenerators (PENG), TENG, and capacitive sensors are four types of flexible sensors used to track body motions [17]. The PENG uses piezoelectric effects and electrostatic induction to monitor signals [18], whereas piezoresistive sensors follow body movement signals by analyzing changes in resistance [19]. The former has a long lifespan, and the latter responds quickly. Based on variations in capacitance, capacitive sensors are used to detect motion [20]. TENG is usually made of two parallel electrodes with a triboelectric layer positioned between them. When pressure is applied, the TENG effect and electrostatic induction cause the electrodes to create current or voltage signals [19]. TENG uses triboelectricity, also known as static electricity, to convert everyday mechanical energy sources into electrical power. In a TENG, electric charges are generated on the contact surfaces when two material surfaces come into contact and then separate, creating an electrical potential between them. The resulting alternating potential from these dynamic mechanical motions can charge batteries or power electronic devices like wireless sensors. Additionally, the potential or current profile produced by a TENG can function as a sensor to monitor motion or detect various chemicals [21]. As shown in Figure 4a, Ding et al. developed a sleep monitoring belt by using a CNT-doped porous TENG. The belt, consisting of two TENG parts in parallel with independent signal pathways, is placed under the chest to monitor breath and heartbeat signals (Figure 4b). The TENG belt structure is shown in Figure 4c. The ballistocardiogram shows real-time voltage output over 30 s, distinguishing low-frequency breath signals and higher-frequency heartbeat signals (Figure 4d). The tester’s heartbeat rate was around 70 beats per minute, with breath cycles of approximately 2 s for inhalation and 5 s for exhalation. Figure 4e shows an enlarged single heartbeat signal with detailed characteristics, including two peaks per cycle, indicating venous and arterial blood pumping, separated by 0.12 s. It used aluminum as the triboelectric pair with PDMS because it is flexible and easy to clip. Figure 4d shows the symmetrical structure of the TENG with aluminum, porous PDMS, textile electrodes, and polyimide layers. When PDMS and aluminum make contact, charges transfer due to their differing triboelectric properties: PDMS becomes negatively charged and aluminum positively charged, maintaining balance. Upon separation, this balance is disrupted by the electrical potential difference. PDMS retains its surface charge, restoring balance once separated [22].

**Figure 5:**
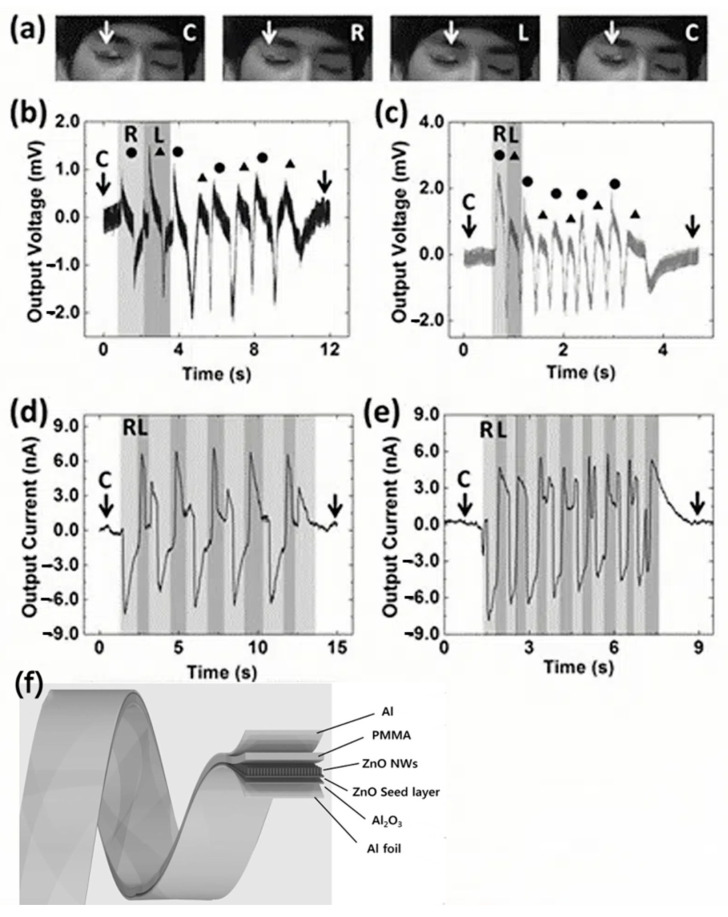
The application of a piezoelectric nanogenerator as an active sensor for measuring eyeball movement during sleep [24]. (**a**) The PENG on the right eyelid was activated by moving the eyeball from right R to center C to left L or from left L to center C to right R. (**b**) The output voltage was detected with slow eye movement. (**c**) The output voltage was detected with rapid eye movement. (**d**) The output current was detected under slow eye movement. (**e**) The output current was detected under rapid eye movement. (**f**) A schematic diagram of the super-flexible PENG. An anodic aluminum oxide (AAO) layer with nanometer pores was fabricated on both surfaces of an Al foil electrode. A ZnO seed layer was sputtered on the AAO surface, followed by ZnO nanowire (NW) growth via a hydrothermal process. Furthermore, during the hydrothermal process, there was minimal variation in their respective thermal expansions, allowing for the fabrication of a mechanically stable PENG device. The ZnO NWs grew densely on the ZnO seed-coated AAO layer, creating a mechanically stable PENG due to similar thermal expansion properties. A thin layer of PMMA was spun-coated on the ZnO NWs to transmit bending forces, and an Al layer with 50 nm thickness was sputtered on the PMMA as the top electrode.

The piezoelectric effect is the ability of certain materials to generate an electric charge when mechanical stress is applied. Electrons in those materials can be forced out of orbit in the force’s direction. Positive and negative charges are built up on opposing sides of the material because of electrons leaving one side and accumulating on the other. The electrons re-enter their orbits when the pressure is removed. When subjected to bending or twisting strain, different materials will behave differently. The direct piezoelectric effect describes the effect from stress to voltage. In the direct piezoelectric effect, mechanical stress, such as compression, stretching, or bending, alters the distribution of electrons within the material. This displacement of electrons creates an electrical potential difference, generating a voltage across the material. For instance, applying a force to a piezoelectric crystal like quartz can produce a measurable voltage [23]. Lee et al. used PENG as an active skin sensor to track eyeball movements, as shown in Figure 5a [24]. The high sensitivity of the super-flexible PENG allowed it to measure slight deformations on the eyelid caused by eye movements, which are linked to sleep patterns (NREM and REM) and brain activity. To demonstrate this, a 3 mm × 10 mm PENG was attached to the right eyelid using eyelash glue, with the electrode facing down for higher sensitivity. The PENG was tested by moving the eye from side to side, generating an alternating signal with peaks corresponding to the eye’s movement. Slow eye movements (~0.4 Hz) produced four-cycle signals (Figure 5b), while rapid movements (~1.6 Hz) produced five-cycle signals (Figure 5c). Output current measurements also indicated eye movements, but signals overlapped due to the response time, as shown in Figure 5d,e. Each one-cycle alternating signal in the output current could not be distinctly obtained during either rapid or slow eye movements. When the eyeball moved side to side, some signals showed one-cycle alternating patterns, while others showed either a positive or negative peak. This overlap occurred because the signal from one direction was covered by the subsequent motion in the opposite direction due to the response time of the output current. Thus, the output voltage can be effectively utilized to detect the eyeball motion for monitoring sleep patterns, tiredness, and possible brain activity [24].

**Figure 6:**
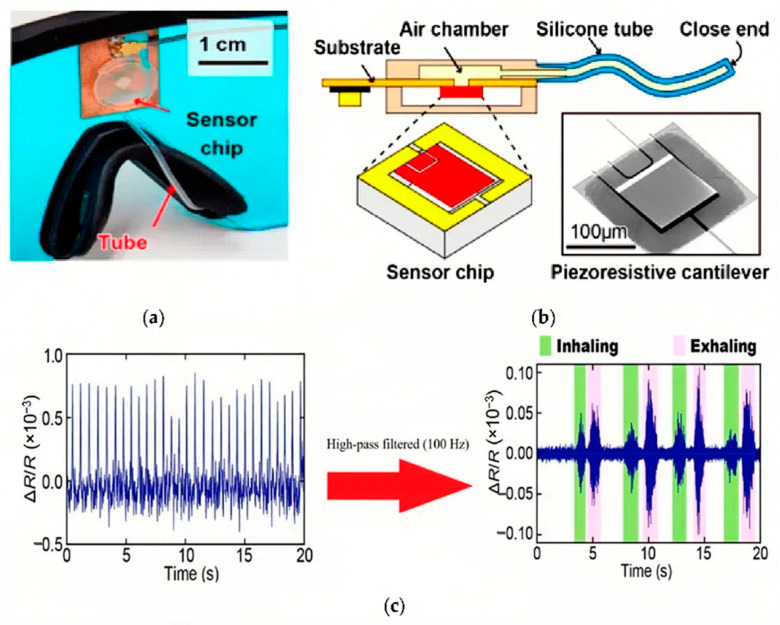
(**a**) The microelectromechanical system (MEMS)-based pressure sensor was mounted on a pair of eyeglasses. (**b**) The schematic representation of the piezoresistive sensor. (**c**) Output signal includes both pulse wave and respiration component (left) after being filtered by a 100 Hz high-pass to extract the breathing component (right) [25]. This device is easy to mount and comfortable, allowing for simultaneous measurement of heart rate and respiratory rate during sleep using a single piezoresistive sensing element. The heartbeat and breathing cause changes in the tube’s inner pressure due to skin vibrations transmitted to the eyeglasses’ nose pad. To measure these pressure changes during sleep, a cantilever was placed in an air chamber connected to a silicon tube, making the sensor sensitive to internal pressure variations.

Piezoresistance is the theory behind piezoresistive sensors, which states that pressure from body motions may change a material’s internal structure. Due to this modification, there is a rise in electrical resistance when the sensor’s cross-sectional area is reduced. The resistance, on the other hand, returns to baseline when the pressure is released. These sensors are perfect for spotting quick motions because of their quick reaction times and straightforward, low-cost construction. However, precise readings must be calibrated because of their susceptibility to temperature variations, and the possibility of residual resistance following pressure release might introduce mistakes. Wang et al. used a piezoresistive array-based force sensor for sleep monitoring, as shown in Figure 6 [25].

**Figure 7:**
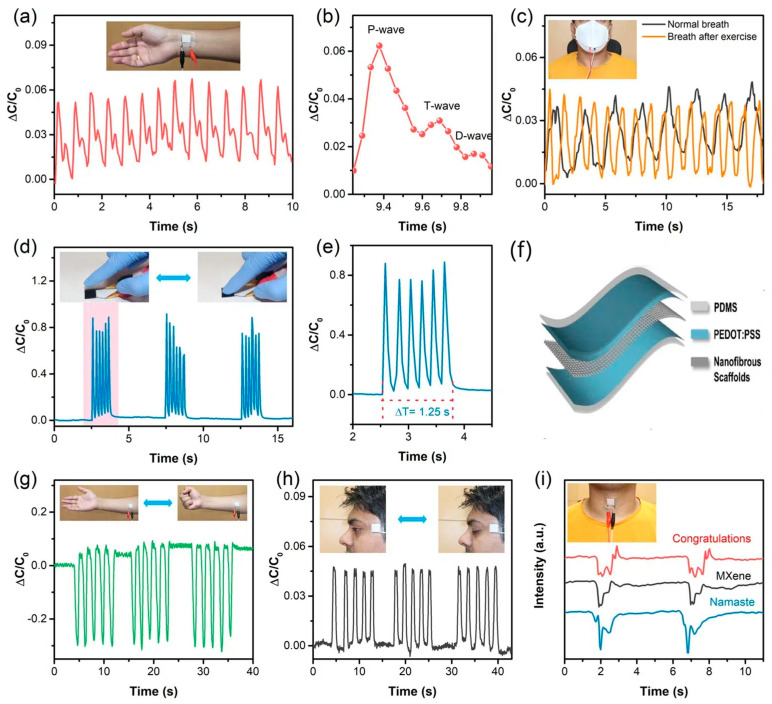
Application of capacitive sensors for real-time and continuous monitoring of muscle movement. (**a**) Real-time pulse monitoring with a sensor on the wrist dermal area. (**b**) Single pulse waveform details. (**c**) Respiration monitoring pre- and post-exercise with mask sensor. (**d**) Detecting early Parkinson’s at 4.8 Hz by mimicking finger knocking. (**e**) Detailed view of 4.8 Hz tapping. The schematic of the capacitive pressure sensor fabrication method involves blending MXene with poly vinylidene fluoride-trifluoroethylene (PVDF-TrFE) to create composite nanofibrous scaffolds (CNS) as a dielectric material through electrospinning. (**f**) The CNS was sandwiched between polystyrene sulfonate (PEDOT)/polydimethylsiloxane (PDMS) films. PEDOT was cross-linked with divinyl sulfone (DVS) for mechanical robustness and stretchability, and each film was post-treated with dimethyl sulfoxide (DMSO) (**g**) Monitoring muscle movement with the sensor on the arm. (**h**) Eye twitch monitoring with the sensor on the eye dermal area. (**i**) Recognizing phonation with the sensor on the superficial dermal layer of the throat [20]. Reprinted (adapted) with permission from ACS Appl. Mater. Interfaces 2020, 12, 19, 22212–22224. Copyright 2020 American Chemical Society.

Capacitive sensors are based on the concept of capacitance, which is a measurement of a capacitor’s capacity to hold electrical charge. These sensors function by having two parallel electrodes separated by a flexible insulator. When body movements alter the distance between the electrodes or the insulator’s properties, the capacitance of the sensor changes. This allows for highly sensitive detection of even subtle movements during sleep thanks to the sensor’s ability to pick up on minute variations. However, their design and fabrication can be more complex compared to piezoresistive sensors, and they are susceptible to external noise from factors like humidity, potentially affecting their accuracy [26]. Sharma et al. used capacitive sensors for real-time and continuous monitoring of muscle movement signals, as shown in Figure 7 [20]. As shown in Figure 7g, the capacitance change sharply increases in the reverse direction (negative ΔC/C_0_) due to ventral arm muscle contraction. The composite nanofibrous scaffold-based sensor effectively detects ocular muscle vibrations during eye twitching (Figure 7h). The insects show the sensor near a volunteer eye. Although the vibration is subtle, resulting in a smaller capacitance change compared to larger deformations, abnormal eyelid spasms can indicate serious brain and nerve disorders.

A summary of the operation mechanisms of the four types of flexible sensors and their responses to external stimuli is shown in Table 2.

## 3. Materials for Flexible Sensors for Body Movement Monitoring

To enable a smooth connection with the human body, flexible, biocompatible, and light materials are required for the sensors. Stretchable conductive polymers and elastomers are often-utilized materials that enable sensors to follow the curves of the body while retaining electrical conductivity [27,28,29,30]. In addition, for wearable integration, new materials with conductive threads or fibers integrated provide a breathable and pleasant alternative. Biocompatible materials, such as medical-grade and silicone adhesives, provide long-term skin friendliness and wearability for biometric and physiological monitoring. Furthermore, a strong performance requires materials that tolerate environmental conditions such as temperature and moisture fluctuations [31,32]. Wearable technology is constantly evolving due to ongoing research and careful selection of suitable materials, which opens a world of possibilities for applications in fitness, healthcare, and other fields [33]. The materials used to manufacture flexible sensors are often divided into three basic categories: polymers, carbon-based materials, and metal-based materials.

Polymers play a foundation role in the development of sensors. For instance, polydimethylsiloxane (PDMS) is favored for its user-friendliness, biocompatibility, and suitability as a base material. Another chosen polymer is polyimide (PI), known for its ability to withstand temperatures, making it ideal for demanding environments [34,35,36]. Enhancing sensors can involve integrating materials into a polymer framework [37]. A good example is the work of Rycewicz and colleagues, who utilized boron-doped diamond nanosheets (BDDNS) on a Kapton substrate (a type of PI) to design a strain sensor for low-strain applications [38].

Metals are the most widely used conductors in sensors. Flexible sensors can be crafted from various metals, including copper, gold, and silver, in different forms like thin films, nanomaterials, and liquid metals. While these metals offer excellent conductivity, some considerations exist. For example, though they are commonly used in electrodes, thin metallic films can have a low signal-to-noise ratio [39]. Liquid metals like EGaIn are a promising alternative due to their flexibility, printability, and biocompatibility, making them suitable electrode materials [40,41]. Metal oxides like zinc oxide nanowires (ZnO NWs) are another area of exploration due to their favorable properties like tunability, affordability, and durability [42,43,44]. MXenes, a new class of metal carbide compounds, are also being investigated for their potential in flexible sensors because of their unique properties, such as high conductivity, mechanical strength, and chemical stability [45,46]. Barium titanate (BaTiO_3_) is another inorganic material with a high dielectric constant and piezoelectric properties, enhancing charge generation and storage capabilities.

Carbon-based materials are popular for their conductivity, lightweight nature, and flexibility, making them ideal for flexible sensors. Common examples include carbon black, carbon nanotubes (CNTs), and graphene. These materials are usually integrated with polymers to create conductive composites [47]. For instance, carbon black mixed with a silicone elastomer can create a sensor with a high gauge factor, stretchability, and repeatability [48,49]. Graphene, known for its exceptional conductivity, can be used in various forms. Graphene oxide, a derivative of graphene, offers printability and allows for fine-tuning of the sensor’s sensitivity through adjustments to its microstructure [50,51,52,53,54]. The above features make graphene-based sensors suitable for wearable health monitoring applications.

## 4. Enhancing Flexibility of Sensors

The flexibility of the sensor is crucial for accurate and comfortable data collection throughout the night. When sensors are flexible, they can easily adjust to the body’s shape, allowing them to detect body movements, even the slightest ones. This ability contributes to the evaluation of sleep quality.

### 4.1. Material Selection

The inherent properties of sensor materials significantly impact their flexibility. Key factors to consider include the modulus of elasticity, elongation at break, and tear strength. Lower modulus values indicate higher flexibility, signifying the material’s reduced stiffness and resistance to deformation. Elongation at break measures the maximum strain a material can endure before breaking, with higher values being desirable for flexible sensors. High tear strength ensures that the material resists tearing or crack propagation under strain, which is crucial for maintaining sensor functionality during deformation. Specific material choices will depend on each sensor design and the desired functionality. There are some material categories commonly used for TENG, PENG, piezoresistive and capacitive sensor shown in Table 3, Table 4, Table 5 and Table 6 as examples.

### 4.2. Structural Design Optimization

The process of optimizing the structural design involves enhancing the performance, flexibility, and durability of the sensors through various design strategies.

**Figure 8:**
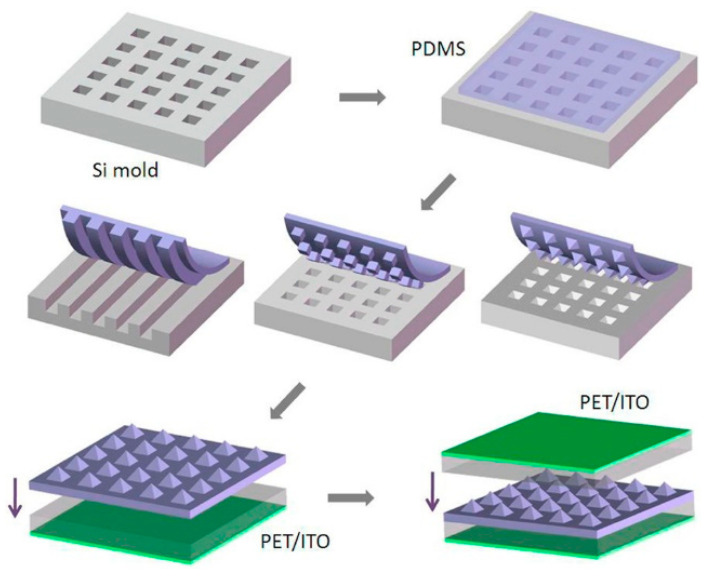
Fabricated patterns on the polymer surfaces to enhance the triboelectric output power. Patterned (100) silicon wafers are used as molds to fabricate PDMS thin films with features such as lines, cubes, and pyramids. The TENG sensor device consists of a layer of patterned PDMS thin film sandwiched between two PET membranes coated with indium tin oxide (ITO) [73]. Adapted with permission from Nano Lett. 2012, 12, 6, 3109–3114. Copyright 2012 American Chemical Society.

Micropatterning/Nanostructuring: the flexible sensor with a micropatterned PDMS surface with microfluidic channels. These channels enhance the sensor’s sensitivity to pressure changes by concentrating the applied force on specific areas. The advantage is that the increased surface area improves the ability of the sensor to detect subtle pressure variations, leading to higher sensitivity for body movements. Fan et al. fabricated patterns on the polymer surfaces to enhance the triboelectric output power, as shown in Figure 8 [73]. To increase triboelectric power output, they used fabricated patterned PDMS films instead of using flat polymer sheets. Si wafer molds with recessed features (lines, cubes, pyramids) were made using photolithography and etching. Molds were treated with trimethylchlorosilane to prevent sticking. Liquid PDMS was mixed, degassed, spin-coated on the molds, cured, and peeled off, resulting in patterned films. The PDMS film was then fixed on an indium tin oxide-coated polyester substrate with a PDMS bonding layer, forming a sandwich structure with another indium tin oxide-coated polyester film. This technique, illustrated in Figure 8, allows for hundreds of replicas from one mold. Metal molds can replace silicon for better durability. The process is simple, low-cost, scalable, and suitable for large-scale production and practical applications.

**Figure 9:**
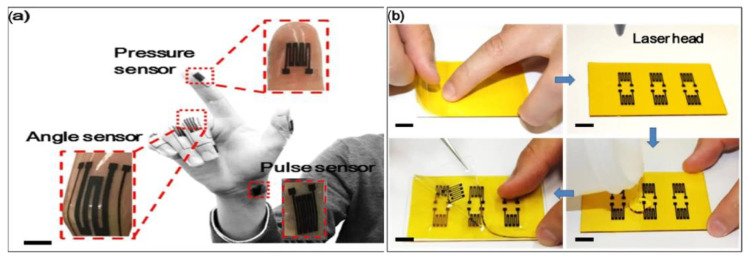
An example of stretchable interconnects designed strain sensors [74]. (**a**) Strain sensors include finger bending strain sensors, a pulse sensor, and a finger pressure sensor with line segments connected in a serpentine structure for cumulative resistance change. (**b**) Sensors’ flexibility with repeated twisting and folding.

Stretchable interconnects: This type of wearable flexible strain sensor uses serpentine-shaped interconnects made from a conductive elastomer. This unique design allows the connections to stay intact during body movements, ensuring electrical flow for monitoring sleep patterns. The benefit of these interconnects is their ability to prevent shorts and disruptions in signals caused by the sensor changing shape while in motion, thus guaranteeing reliable and precise data collection. Wu et al. used the serpentine-shaped sensor design to prevent signal disruptions, as shown in Figure 9 [74]. In Figure 9, the fabrication process of strain sensors is illustrated. A thin Kapton tape measuring 50 μm in thickness (with a silicone layer 25 μm in thickness and a polyimide layer 25 μm in thickness) is affixed to a glass base. The carbon-based materials (graphite or graphene) are converted from polyimide by using a CO_2_ laser. Serpentine structures are employed for sensing purposes. A serpentine structure with three cycles is employed for strain sensors, while an eight-cycle structure is used for pressure sensors to enhance sensitivity. Figure 9 showcases the flexibility of these sensors to highlight their ability to twist and fold.

**Figure 10:**
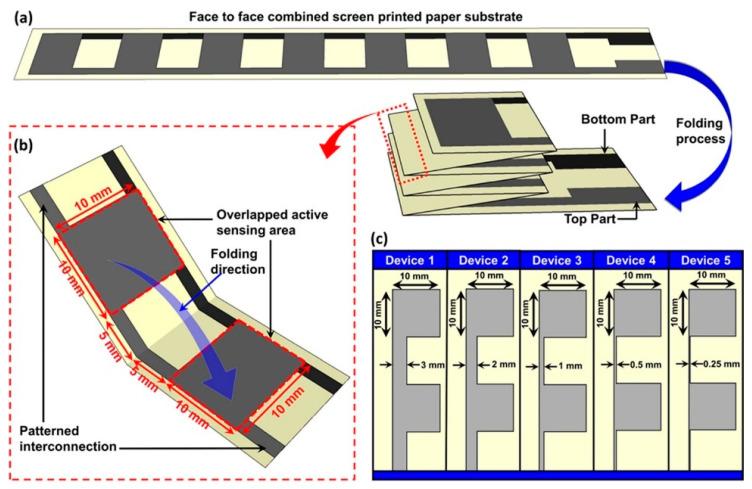
An origami-based tactile sensor crafted from a paper substrate and coated with graphene is used to detect bruxism and neck posture. (**a**) Schematic diagram of 6-fold patterned tactile sensors. (**b**) Highlights show the folded location, showing interconnection patterns and overlapping sensing regions. (**c**) Layouts with line widths scaled down from 3 mm to 0.25 mm [75]. Reprinted (adapted) with permission from ACS Appl. Mater. Interfaces 2024, 16, 3, 4231–4241. Copyright 2024 American Chemical Society.

Meshed structures: A flexible sensor with a meshed design on its PDMS layer can allow for improved flexibility while maintaining good pressure sensitivity. The mesh openings also enhance breathability for wearable applications. Wu et al. proved that the breathability and hydrophobicity of the meshed structure sensor design play an important role in sweat resistance and wearing comfort in the long term [65]. Origami-inspired design: A strain sensor with an origami-inspired folded structure can allow for high stretchability while maintaining good electrical conductivity. This design is well-suited for tracking large changes in shape that occur during physical activities. One key benefit is that structures inspired by origami offer flexibility and stretchiness, making them perfect for sensors utilized in activities with high movement. Kamakar et al. used an origami-based tactile sensor crafted from a paper substrate and coated with graphene, as shown in Figure 10 [75]. These sensors measure tactile information through changes in electrical contact resistance (ECR) between upper and lower substrates under pressure. The folding process improves the contact areas, enhancing the sensor’s sensitivity to detect the changes. The connections at the folds play an important role in the performance and help optimize sensitivity. These sensors have applications, such as wearables, that can detect teeth grinding (bruxism) and monitor the neck postures during sleep.

Meshed patterns found in sensors can be utilized in conjunction with TENG, PENG, piezoresistive sensors, and capacitive sensors. These designs have enhanced flexibility, breathability, and resistance to water, making them ideal for applications that prioritize comfort and prolonged usage. The mesh apertures improve the sensor’s ability to adapt to body movements while maintaining sensitivity. In the realm of sleep monitoring, sensors incorporating mesh structures can capture signals, including pressure and strain signals. Pressure signals: TENG, capacitive sensors can detect these signals to track body movements, changes in sleeping positions, and pressure points, which is essential for evaluating comfort levels and identifying issues like pressure sores. Strain signals: PENG and piezoresistive sensors are capable of monitoring breathing patterns as well through chest expansion and contraction to aid in detecting respiratory problems and ensuring optimal sleep quality [75].

### 4.3. Combination of Materials

**Figure 11:**
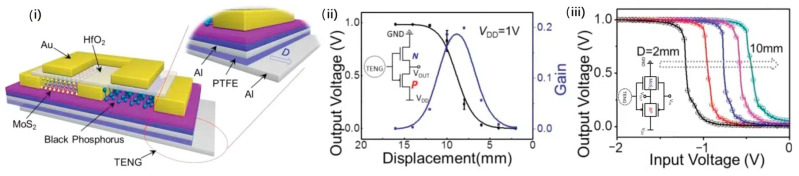
PTFE and aluminum combination. (**i**) A tenable tribotronic dual-gate logic device using a sliding TENG with PTFE and aluminum. (**ii**) TENG-generated voltage drives molybdenum disulfide and black phosphorus field-effect transistors for logic output. (**iii**) Verified self-driven threshold voltage adjustment by spacing between aluminum and PTFE layers [76].

While the properties of individual materials are important, studying the synergy of multiple materials could be a possibility for enhancing the flexibility of sensors in sleep body movement monitoring. An example is the combination of polytetrafluoroethylene (PTFE) and aluminum used in TENG sensors, as shown in Figure 11. PTFE’s inherent flexibility makes it ideal for wearable sensors, allowing them to adapt to various body postures and movements during sleep without compromising performance. This pairing leverages their contrasting triboelectric charges (PTFE negative, aluminum positive) to generate a high triboelectric coefficient, efficiently converting movements into electricity. The combination boasts a high triboelectric coefficient, efficiently generating electricity from even small movements. Figure 11ii,iii shows a tuneable tribotronic logic device based on this TENG. The generated triboelectric voltage acts as the gate voltage for a logic unit, eliminating the need for an external power source. This self-powered device boasts an impressive performance, with an on/off ratio exceeding 106 and a cutoff current below 1 pA/μm. Furthermore, improvements were achieved in adjustable gain (≈13.8) and power consumption (≈1 nW) [76]. Additionally, both PTFE and aluminum are relatively durable materials, ensuring long-term functionality in sleep monitoring sensors.

**Figure 12:**
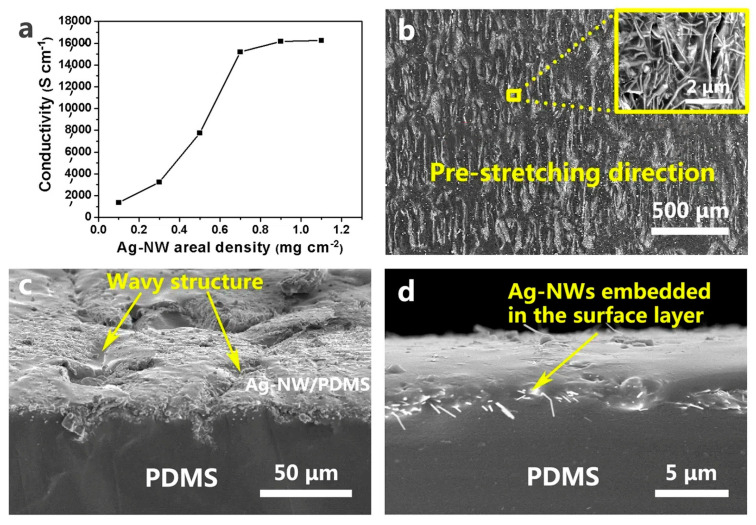
PDMS and silver nanowires combination. (**a**) Conductivity of the PSPE circuit as a function of Ag-NW areal density; (**b**) top-view SEM image of the PSPE circuit surface, with the inset showing a magnified view of the Ag-NWs; (**c**) side-view SEM image of the PSPE circuit surface; and (**d**) SEM image of the PSPE circuit cross-section. Reused and modified with permissions after [77].

The PDMS and silver nanowire (Ag-NW) combination is also a good example, as shown in Figure 12. PDMS offers excellent flexibility and biocompatibility, making it ideal for comfortable wear throughout the night. Silver nanowires, with their unique structure, can be shaped into flexible electrodes or interconnected, creating conductive pathways that bend and stretch without breaking. This combination can enhance conductivity and promote durability, ensuring reliable data collection and comfortable wear throughout the night during sleep. Figure 12a shows the conductivity of the circuit as a function of the Ag-NW areal density. The percolation threshold appears at 0.7 mg cm^−2^, with a corresponding electrical conductivity of 1.52 × 10^4^ S cm^−1^, significantly higher than the 8.13 × 10^3^ S cm^−1^ reported for stretchable Ag-NW/PDMS circuits. This conductivity meets the requirements for various electronic applications, and is comparable to that of universal Sn/Pb eutectic solders (~3 × 10^4^ S cm^−1^). Therefore, 0.7 mg cm^−2^ of Ag-NWs is selected for fabricating stretchable circuits. Figure 12b shows the SEM image of the top surface of the Ag-NW/PDMS nanocomposite circuit with a wavy pattern in the stretching direction. The wavy structure is key to the circuit’s high electrical stability upon stretching. Figure 12c provides a magnified SEM image, clearly showing the wavy structure. Figure 12d reveals Ag-NWs buried in the PDMS surface layer, forming a conductive layer on the top surface [77].

Graphene and polyethylene terephthalate can greatly enhance their conductivity, strength, and energy-harvesting capabilities. The remarkable flexibility of graphene makes it an ideal choice for sensors. Shankaregowda et al. [78] utilized this combination in the experiment, demonstrating that the sensor exhibits conductivity with an optical transmittance of 97%. By utilizing chemical vapor deposition-grown graphene as a friction surface, the output efficiency was significantly enhanced, resulting in a peak output of 650 V, 12 μA, and 3.28 mW at 60 MΩ load resistance. This innovative device produces energy, making it a reliable power source for flexible electronics.

## 5. Future Trends in Flexible Sensor Technology for Sleep Body Movement Monitoring

### 5.1. Innovative Materials and Structural Designs

**Figure 13:**
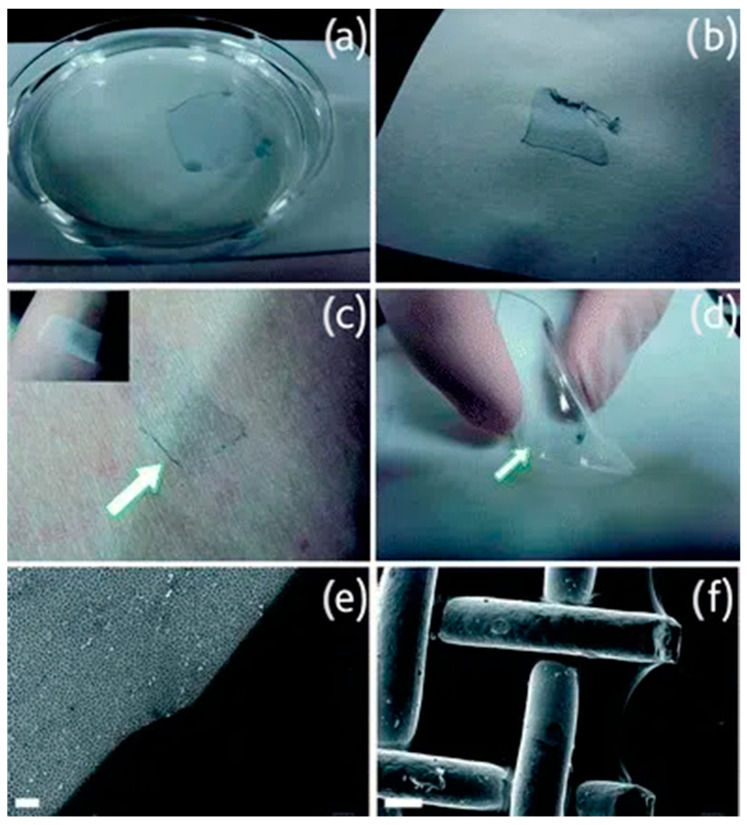
An example of using large-area, free-standing, ultra-thin films of the conductive polymer PEDOT/PSS, which enables imperceptible integration into wearable sensors for sleep monitoring. (**a**) Floating in water after PVA dissolves. (**b**) Nanofilms collected onto paper. (**c**) Nanofilms collected onto the skin. (**d**) Nanofilms collected onto flexible PDMS. (**e**) Nanofilms collected onto porous alumina (2 μm scale). (**f**) Nanofilms collected onto steel mesh (100 μm scale) [80].

The future of flexible sensor technology for sleep monitoring presents exciting possibilities. Enhanced flexibility and wearability will be achieved through the continuous exploration of innovative materials and structural designs, creating even more flexible and comfortable sensors. Trends include self-healing polymers that autonomously repair minor damage, extending the sensor’s lifespan and improving user comfort [79]. Greco et al. used the supporting layer technique to develop large-area, free-standing, ultra-thin films of the conductive polymer poly(3,4-ethylenedioxythiophene)/poly(styrenesulfonate) (PEDOT/PSS), offering a fast and reliable method to produce conductive nanofilms that can be released in water and collected onto various substrates while retaining their functional properties, which enables imperceptible integration into wearable sensors during sleep, as shown in Figure 13 [80]. Moreover, the integration of layouts that include channels into sensors offers advanced functionalities through microfluidic design, such as analyzing sweat or delivering drugs to specific areas while asleep [81].

### 5.2. Integration with Artificial Intelligence and Machine Learning

Artificial intelligence and machine learning integration are paving the way for the future of sleep monitoring. By utilizing these technologies, we can delve deeper into sensor data to gain insights. Their algorithms are capable of analyzing sensor data to provide feedback on sleep quality, pinpoint potential sleep disruptions, and recommend lifestyle adjustments for better sleep outcomes [82]. Moreover, machine learning algorithms can be trained to detect subtle changes in sleep patterns that may indicate underlying health issues, like sleep apnea or restless leg syndrome [83]. By focusing on these trends, researchers can work towards creating sensors that offer a more comfortable, personalized, and data-driven approach to monitoring sleep.

## 6. Conclusions

Flexible sensors, with their lightweight and conformable nature, enable seamless integration into wearable devices, which provide continuous and non-intrusive monitoring of body movements during sleep. This review comprehensively explores advancements in flexible sensors for sleep monitoring, which include working principles, materials, strategies for enhanced flexibility, applications, and future trends. The choice of sensor materials focuses on flexibility and durability. Material selection and structural design optimization are important in achieving optimal sensor performance. Combining materials with synergistic properties such as PDMS for comfort and silver nanowires for conductivity is also a proven method to improve the performance of sensors. The future of flexible sensor technology for sleep monitoring is coming with exciting possibilities. Continuous advancements in materials and design will lead to even more comfortable and user-friendly sensors. Furthermore, integration with artificial intelligence and machine learning will enhance the early detection of sleep disorders and promote better overall health. By addressing the remaining challenges and embracing these trends, flexible sensors have the potential to revolutionize sleep monitoring.

## Figures and Tables

**Table 1:** The body movements characteristic of each sleep stage [6].

	NREM/N1	NREM/N2	NREM/N3	REM
Description	Transition from wakefulness to sleep.	Further decrease in body temperature. Slowing of breathing and heart rate.	Muscle tone, heart rate, and breathing reach their lowest points.	Rapid eye movements under closed eyelids. Most dreaming occurs.
Body Movements	Brief muscle twitches or a slight change in body position.	Less frequent and less pronounced compared to NREM N1. Occasional turns or shifts in position.	Minimal body movements. Might experience occasional positional adjustments, but overall is quite still.	Eye movements. Muscle activity in the rest of the body is generally inhibited.
Normal Length	1–7 min	10–25 min	20–40 min	10–60 min

**Table 2:** The operation mechanisms of the four types of flexible sensors [17,18,19,20].

Sensor Type	Operation Mechanism	Response to Stimuli
TENG	Triboelectric effect and electrostatic induction cause electrodes to generate current/voltage signals when pressure is applied	Generates electric charges on contact surfaces when two materials come into contact and separate, creating an electrical potential
PENG	Piezoelectric effect generates an electric charge in response to mechanical stress	Mechanical stress alters electron distribution within the material, creating an electrical potential difference
Piezoresistive	Changes in electrical resistance due to pressure from body motions altering the material’s internal structure	Pressure reduces the sensor’s cross-sectional area, increasing electrical resistance; resistance returns to baseline when pressure is released
Capacitive	Changes in capacitance due to body movements altering the distance between electrodes and the insulator properties	Body movements alter the capacitance of the sensor by changing the distance between electrodes or the insulator’s properties, allowing for sensitive detection of even subtle movements during sleep

**Table 3:** The materials commonly used for TENG sensors.

Materials	Properties	Key Flexibility Factors	Application
PDMS	Good biocompatibility, ease of processing, and tenable mechanical properties [55]	Low modulus, high elongation at break, good tear strength	Contact layers
Silicone Rubber	Flexibility, biocompatibility, and electrical insulation [56,57]	Low modulus, high elongation at break, good tear strength	Contact layers
Liquid metals	Like Galinstan (EGaIn), they exhibit excellent flexibility due to their liquid nature and enable the forcing of stretchable sensor designs [58]	High flexibility, low modulus	Electrodes
Conductive polymers	Like polyaniline (PANI) and polypyrrole (PPy), they can be tailored for flexibility through doping or structural modifications [59]	Tuneable flexibility, high conductivity	Electrodes
Graphene	Provides exceptional flexibility, conductivity, and mechanical strength, making it a promising electrode material for flexible sensors [60]	Exceptional flexibility, high conductivity, high strength	Electrodes
Flexible Polymers	Bend and deform without compromising device performance [61]	Low modulus, high elongation at break	Substrates
Textile Fabrics	Provide additional flexibility and breathability [62]	High flexibility, breathability	Substrates

**Table 4:** The materials commonly used for PENG sensors.

Materials	Properties	Key Flexibility Factors	Application
Zinc Oxide (ZnO)	High piezoelectric coefficient, easy to fabricate [63]	Nanowire flexibility, compatibility with flexible substrates	Electrodes
Barium Titanate (BaTiO_3_)	High piezoelectric and dielectric properties [64]	Ceramic flexibility in thin films, compatibility with polymers	Electrodes
Polyvinylidene Fluoride (PVDF)	Flexible, high piezoelectric response [65]	Polymer flexibility, mechanical durability	Transducer layer
Lead Zirconate Titanate (PZT)	High piezoelectric properties, robust [66]	Thin-film deposition, mechanical stability	Transducer layer
Aluminium Nitride (AlN)	High thermal conductivity, stability [67]	Thin-film fabrication, mechanical flexibility	Transducer layer

**Table 5:** The materials commonly used for piezoresistive sensors.

Materials	Properties	Key Flexibility Factors	Application
Silicone Rubber	Flexibility, biocompatibility, and electrical insulation [56,57]	Low modulus, high elongation at break, good tear strength	Substrate
Carbon Nanotubes (CNT)	High flexibility, excellent conductivity [68]	High tensile strength, flexibility	Transducer layer
Polypyrrole	Good conductivity, flexible, biocompatible [69]	Biocompatibility, mechanical flexibility	Electrodes
Graphene	High strength, flexibility, and conductivity [60]	Ultra-thin structure, mechanical flexibility	Electrodes

**Table 6:** The materials commonly used for capacitive sensors.

Materials	Properties	Key Flexibility Factors	
Silicone Rubber	Flexibility, biocompatibility, and electrical insulation [56,57].	Low modulus, high elongation at break, good tear strength	Substrate
Polyimide	High flexibility, good thermal stability [70]	Flexibility, thermal stability	Substrate
PDMS	Good biocompatibility, ease of processing, and tenable mechanical properties [55].	Low modulus, high elongation at break, good tear strength	Substrate
Aluminium Oxide (Al_2_O_3_)	High dielectric strength, stability [71]	Thin-film deposition, mechanical stability	Transducer layer
Polyvinylidene Fluoride (PVDF)	Good flexibility, high dielectric constant [72]	Flexibility, mechanical durability	Transducer layer

## References

[1] Chokroverty S. (1994). 2—An overview of sleep. Sleep Disorders Medicine.

[2] World Health Statistics (2017). Monitring Health for the SDGs Sustainable Development Goals.

[3] Lenfant C. (1998). Task Force on Behavioral Research in Cardiovascular, Lung, and Blood Health and Disease. Circulation.

[4] Patel A.K., Reddy V., Shumway K.R., Araujo J.F. (2024). Physiology, sleep stages. StatPearls.

[5] Imtiaz S.A. (2021). A Systematic Review of Sensing Technologies for Wearable Sleep Staging. Sensors.

[6] “Your Guide to Healthy Sleep|NHLBI, NIH,” (in en). https://www.nhlbi.nih.gov/resources/your-guide-healthy-sleep.

[7] Cay G., Ravichandran V., Sadhu S., Zisk A.H., Salisbury A.L., Solanki D., Mankodiya K. (2022). Recent Advancement in Sleep Technologies: A Literature Review on Clinical Standards, Sensors, Apps, and AI Methods. IEEE Access.

[8] Danilenko K.V., Stefani O., Voronin K.A., Mezhakova M.S., Petrov I.M., Borisenkov M.F., Markov A.A., Gubin D.G. (2022). Wearable Light-and-Motion Dataloggers for Sleep/Wake Research: A Review. Appl. Sci..

[9] Sannino G., De Falco I., Bhoi A.K., Mallick P.K., Mohanty M.N., Albuquerque V.H.C.D. (2021). Use of machine learning algorithms to identify sleep phases starting from ECG signals. Hybrid Artificial Intelligence and IoT in Healthcare.

[10] Clemens S., Zigmond M.J., Wiley C.A., Chesselet M.-F. (2023). Chapter 37—Restless Legs Syndrome. Neurobiology of Brain Disorders.

[11] Kogan D., Jain A., Kimbro S., Gutierrez G., Jain V. (2016). Respiratory Inductance Plethysmography Improved Diagnostic Sensitivity and Specificity of Obstructive Sleep Apnea. Respir. Care.

[12] Virkkala J., Hasan J., Värri A., Himanen S.-L., Müller K. (2007). Automatic sleep stage classification using two-channel electro-oculography. J. Neurosci. Methods.

[13] Song W., Gan B., Jiang T., Zhang Y., Yu A., Yuan H., Chen N., Sun C., Wang Z.L. (2016). Nanopillar Arrayed Triboelectric Nanogenerator as a Self-Powered Sensitive Sensor for a Sleep Monitoring System. ACS Nano.

[14] Lin Z., Yang J., Li X., Wu Y., Wei W., Liu J., Chen J., Yang J. (2018). Large-Scale and Washable Smart Textiles Based on Triboelectric Nanogenerator Arrays for Self-Powered Sleeping Monitoring. Adv. Funct. Mater..

[15] Zhang N., Li Y., Xiang S., Guo W., Zhang H., Tao C., Yang S., Fan X. (2020). Imperceptible sleep monitoring bedding for remote sleep healthcare and early disease diagnosis. Nano Energy.

[16] Kou H., Wang H., Cheng R., Liao Y., Shi X., Luo J., Li D., Wang Z.L. (2022). Smart Pillow Based on Flexible and Breathable Triboelectric Nanogenerator Arrays for Head Movement Monitoring during Sleep. ACS Appl. Mater. Interfaces.

[17] Meng K., Xiao X., Wei W., Chen G., Nashalian A., Shen S., Chen J. (2022). Wearable Pressure Sensors for Pulse Wave Monitoring. Adv. Mater..

[18] Huo Z., Wei Y., Wang Y., Wang Z.L., Sun Q. (2022). Integrated Self-Powered Sensors Based on 2D Material Devices. Adv. Funct. Mater..

[19] Zhang F., Yang K., Pei Z., Wu Y., Sang S., Zhang Q., Jiao H. (2022). A highly accurate flexible sensor system for human blood pressure and heart rate monitoring based on graphene/sponge. RSC Adv..

[20] Sharma S., Chhetry A., Sharifuzzaman, Yoon H., Park J.Y. (2020). Wearable Capacitive Pressure Sensor Based on MXene Composite Nanofibrous Scaffolds for Reliable Human Physiological Signal Acquisition. ACS Appl. Mater. Interfaces.

[21] Zazoum B., Batoo K.M., Khan M.A.A. (2022). Recent Advances in Flexible Sensors and Their Applications. Sensors.

[22] Ding X., Cao H., Zhang X., Li M., Liu Y. (2018). Large Scale Triboelectric Nanogenerator and Self-Powered Flexible Sensor for Human Sleep Monitoring. Sensors.

[23] Deng W., Zhou Y., Libanori A., Chen G., Yang W., Chen J. (2022). Piezoelectric nanogenerators for personalized healthcare. Chem. Soc. Rev..

[24] Lee S., Hinchet R., Lee Y., Yang Y., Lin Z., Ardila G., Montès L., Mouis M., Wang Z.L. (2014). Ultrathin Nanogenerators as Self-Powered/Active Skin Sensors for Tracking Eye Ball Motion. Adv. Funct. Mater..

[25] De Fazio R., Stabile M., De Vittorio M., Velázquez R., Visconti P. (2021). An Overview of Wearable Piezoresistive and Inertial Sensors for Respiration Rate Monitoring. Electronics.

[26] Djakow M., Braun A., Marinc A. (2014). MoviBed—Sleep analysis using capacitive sensors. Universal Access in Human-Computer Interaction. Design for All and Accessibility Practice.

[27] Liu H., Wang L., Lin G., Feng Y. (2022). Recent progress in the fabrication of flexible materials for wearable sensors. Biomater. Sci..

[28] Zhu C., Wu J., Yan J., Liu X. (2023). Advanced Fiber Materials for Wearable Electronics. Adv. Fiber Mater..

[29] Wu Y., Ma Y., Zheng H., Ramakrishna S. (2021). Piezoelectric materials for flexible and wearable electronics: A review. Mater. Des..

[30] Liu C., Wang Q., Wang C., Wang Q., Zhao W., He Z., Zheng Y., Jing Y., Sun X., Zhang S. (2023). State of the Art Overview Wearable Biohazard Gas Sensors Based on Nanosheets for Environment Monitoring Applications. Trends Environ. Anal. Chem..

[31] Zhou Y., Zhang Y., Zhou Y., Zhao L., Liu F., Yan X., Sun P., Lu G. (2023). Waterproof breathable multifunctional flexible sensor for underwater tactile sensing and ammonia gas monitoring. Nano Energy.

[32] Bocchetta P., Frattini D., Ghosh S., Mohan A.M.V., Kumar Y., Kwon Y. (2020). Soft Materials for Wearable/Flexible Electrochemical Energy Conversion, Storage, and Biosensor Devices. Materials.

[33] Javaid A., Zulfiqar M.H., Saleem M.S., Khan M.A., Mehmood M.Q., Massoud Y. (2023). Paper-based wearable ultra-sensitive strain sensors for fitness monitoring. Flex. Print. Electron..

[34] Harito C., Utari L., Putra B.R., Yuliarto B., Purwanto S., Zaidi S.Z.J., Bavykin D.V., Marken F., Walsh F.C. (2020). Review—The Development of Wearable Polymer-Based Sensors: Perspectives. J. Electrochem. Soc..

[35] Lin L., Park S., Kim Y., Bae M., Lee J., Zhang W., Gao J., Paek S.H., Piao Y. (2023). Wearable and stretchable conductive polymer composites for strain sensors: How to design a superior one?. Nano Mater. Sci..

[36] Pavel I.-A., Lakard S., Lakard B. (2022). Flexible Sensors Based on Conductive Polymers. Chemosensors.

[37] Geng Y., Cao R., Innocent M.T., Hu Z., Zhu L., Wang L., Xiang H., Zhu M. (2022). A high-sensitive wearable sensor based on conductive polymer composites for body temperature monitoring. Compos. Part A Appl. Sci. Manuf..

[38] Rycewicz M., Ficek M., Gajewski K., Kunuku S., Karczewski J., Gotszalk T., Wlasny I., Wysmołek A., Bogdanowicz R. (2021). Low-strain sensor based on the flexible boron-doped diamond-polymer structures. Carbon.

[39] Hogas I., Fosalau C., Zet C. A new strain sensor based on electrospinning and thin film technologies. Proceedings of the 2016 International Conference and Exposition on Electrical and Power Engineering (EPE).

[40] Dickey M.D., Chiechi R.C., Larsen R.J., Weiss E.A., Weitz D.A., Whitesides G.M. (2008). Eutectic gallium-indium (EGaIn): A liquid metal alloy for the formation of stable structures in microchannels at room temperature. Adv. Funct. Mater..

[41] Won P., Jeong S., Majidi C., Ko S.H. (2021). Recent advances in liquid-metal-based wearable electronics and materials. iScience.

[42] Costa J.C., Spina F., Lugoda P., Garcia-Garcia L., Roggen D., Münzenrieder N. (2019). Flexible sensors-From materials to applications. Technologies.

[43] Yoon Y., Truong P.L., Lee D., Ko S.H. (2022). Metal-Oxide Nanomaterials Synthesis and Applications in Flexible and Wearable Sensors. ACS Nanosci. Au.

[44] Lee T., Lee W., Kim S.W., Kim J.J., Kim B.S. (2016). Flexible Textile Strain Wireless Sensor Functionalized with Hybrid Carbon Nanomaterials Supported ZnO Nanowires with Controlled Aspect Ratio. Adv. Funct. Mater..

[45] Sobolčiak P., Tanvir A., Sadasivuni K.K., Krupa I. (2019). Piezoresistive sensors based on edlectrospun mats modified by 2D Ti_3_C_2_T_x_ MXene. Sensors.

[46] Zhang W., Wang P.-L., Huang L.-Z., Guo W.-Y., Zhao J., Ma M.-G. (2023). A stretchable, environmentally tolerant, and photoactive liquid metal/MXene hydrogel for high performance temperature monitoring, human motion detection and self-powered application. Nano Energy.

[47] Xiang L., Zhang H., Hu Y., Peng L.-M. (2018). Carbon nanotube-based flexible electronics. J. Mater. Chem. C.

[48] Shintake J., Piskarev Y., Jeong S.H., Floreano D. (2018). Ultrastretchable Strain Sensors Using Carbon Black-Filled Elastomer Composites and Comparison of Capacitive Versus Resistive Sensors. Adv. Mater. Technol..

[49] Wang Z., Guan X., Huang H., Wang H., Lin W., Peng Z. (2019). Full 3D Printing of Stretchable Piezoresistive Sensor with Hierarchical Porosity and Multimodulus Architecture. Adv. Funct. Mater..

[50] Jang H., Park Y.J., Chen X., Das T., Kim M., Ahn J. (2016). Graphene-Based Flexible and Stretchable Electronics. Adv. Mater..

[51] Lee S., Jo I., Kang S., Jang B., Moon J., Park J.B., Lee S., Rho S., Kim Y., Hong B.H. (2017). Smart Contact Lenses with Graphene Coating for Electromagnetic Interference Shielding and Dehydration Protection. ACS Nano.

[52] Wang Y., Hao J., Huang Z., Zheng G., Dai K., Liu C., Shen C. (2018). Flexible electrically resistive-type strain sensors based on reduced graphene oxide-decorated electrospun polymer fibrous mats for human motion monitoring. Carbon.

[53] Wang Z.-Q., Lan Y.-S., Zeng Z.-Y., Chen X.-R., Chen Q.-F. (2019). Magnetic structures and optical properties of rare-earth orthoferrites RFeO3 (R = Ho, Er, Tm and Lu). Solid State Commun..

[54] “A graphene roll-out,” (in en), MIT News|Massachusetts Institute of Technology. https://news.mit.edu/2018/manufacturing-graphene-rolls-ultrathin-membranes-0418.

[55] Miranda I., Souza A., Sousa P., Ribeiro J., Castanheira E.M.S., Lima R., Minas G. (2021). Properties and Applications of PDMS for Biomedical Engineering: A Review. J. Funct. Biomater..

[56] Srivastava S.K., Kuila T., Papaspyrides C.D., Kiliaris P. (2014). Chapter 18—Fire Retardancy of Elastomers and Elastomer Nanocomposites. Polymer Green Flame Retardants.

[57] Bigg D.M. (1976). A review of techniques for processing ultra-high modulus polymers. Polym. Eng. Sci..

[58] Chen J., Tian G., Liang C., Yang D., Zhao Q., Liu Y., Qi D. (2023). Liquid metal–hydrogel composites for flexible electronics. Chem. Commun..

[59] Nambiar S., Yeow J.T. (2011). Conductive polymer-based sensors for biomedical applications. Biosens. Bioelectron..

[60] Kim S.J., Choi K., Lee B., Kim Y., Hong B.H. (2015). Materials for Flexible, Stretchable Electronics: Graphene and 2D Materials. Annu. Rev. Mater. Res..

[61] Afshari M., Sikkema D.J., Lee K., Bogle M. (2008). High Performance Fibers Based on Rigid and Flexible Polymers. Polym. Rev..

[62] Grancaric A.M., Tarbuk A., Pusic T. (2005). Electrokinetic properties of textile fabrics. Color. Technol..

[63] Liao Q., Zhang Z., Zhang X., Mohr M., Zhang Y., Fecht H.-J. (2014). Flexible piezoelectric nanogenerators based on a fiber/ZnO nanowires/paper hybrid structure for energy harvesting. Nano Res..

[64] Wada S., Yasuno H., Hoshina T., Nam S.-M., Kakemoto H., Tsurumi T. (2003). Preparation of nm-Sized Barium Titanate Fine Particles and Their Powder Dielectric Properties. Jpn. J. Appl. Phys..

[65] Zheng Z., Wang X., Hang G., Duan J., Zhang J., Zhang W., Liu Z. (2024). Recent progress on flexible poly(vinylidene fluoride)-based piezoelectric nanogenerators for energy harvesting and self-powered electronic applications. Renew. Sustain. Energy Rev..

[66] Khanbareh H., Rasheed A., Khaliq J., Asadi K. (2022). 13—Piezoelectric composites. Organic Ferroelectric Materials and Applications.

[67] Pinto R.M., Gund V., Calaza C., Nagaraja K., Vinayakumar K. (2022). Piezoelectric aluminum nitride thin-films: A review of wet and dry etching techniques. Microelectron. Eng..

[68] Naresh V., Lee N. (2021). A Review on Biosensors and Recent Development of Nanostructured Materials-Enabled Biosensors. Sensors.

[69] Sriprasertsuk S., Mathias S.C., Varcoe J.R., Crean C. (2021). Polypyrrole-coated carbon fibre electrodes for paracetamol and clozapine drug sensing. J. Electroanal. Chem..

[70] Kisić M., Blaž N., Živanov Ž.Č.L., Huđik A. Flexible polyimide based capacitive displacement sensor. Proceedings of the 2017 40th International Spring Seminar on Electronics Technology (ISSE).

[71] Lim G.-H., Kim I.-Y., Park J.-Y., Choa Y.-H., Lim J.-H. (2023). Anodic Aluminum Oxide-Based Chemi-Capacitive Sensor for Ethanol Gas. Nanomaterials.

[72] Qi F., Xu L., He Y., Yan H., Liu H. (2023). PVDF-Based Flexible Piezoelectric Tactile Sensors: Review. Cryst. Res. Technol..

[73] Fan F.-R., Lin L., Zhu G., Wu W., Zhang R., Wang Z.L. (2012). Transparent Triboelectric Nanogenerators and Self-Powered Pressure Sensors Based on Micropatterned Plastic Films. Nano Lett..

[74] Wu Y., Karakurt I., Beker L., Kubota Y., Xu R., Ho K.Y., Zhao S., Zhong J., Zhang M., Wang X. (2018). Piezoresistive stretchable strain sensors with human machine interface demonstrations. Sens. Actuators A Phys..

[75] Karmakar R.S., Huang J.-F., Chu C.-P., Mai M.-H., Chao J.-I., Liao Y.-C., Lu Y.-W. (2023). Origami-Inspired Conductive Paper-Based Folded Pressure Sensor with Interconnection Scaling at the Crease for Novel Wearable Applications. ACS Appl. Mater. Interfaces.

[76] Li Q., Dai K., Zhang W., Wang X., You Z., Zhang H. (2021). Triboelectric nanogenerator-based wearable electronic devices and systems: Toward informatization and intelligence. Digit. Signal Process..

[77] Huang G.-W., Xiao H.-M., Fu S.-Y. (2015). Wearable Electronics of Silver-Nanowire/Poly(dimethylsiloxane) Nanocomposite for Smart Clothing. Sci. Rep..

[78] Shankaregowda S.A., Nanjegowda C.B., Cheng X.-L., Shi M.-Y., Liu Z.-F., Zhang H.-X. (2016). A Flexible and Transparent Graphene-Based Triboelectric Nanogenerator. IEEE Trans. Nanotechnol..

[79] Wang S., Urban M.W. (2020). Self-healing polymers. Nat. Rev. Mater..

[80] Greco F., Zucca A., Taccola S., Menciassi A., Fujie T., Haniuda H., Takeoka S., Dario P., Mattoli V. (2011). Ultra-thin conductive free-standing PEDOT/PSS nanofilms. Soft Matter.

[81] Kim S.B., Lee K., Raj M.S., Lee B., Reeder J.T., Koo J., Hourlier-Fargette A., Bandodkar A.J., Won S.M., Sekine Y. (2018). Soft, Skin-Interfaced Microfluidic Systems with Wireless, Battery-Free Electronics for Digital, Real-Time Tracking of Sweat Loss and Electrolyte Composition. Small.

[82] Goldstein C.A., Berry R.B., Kent D.T., Kristo D.A., Seixas A.A., Redline S., Westover M.B. (2020). Artificial intelligence in sleep medicine: Background and implications for clinicians. J. Clin. Sleep Med..

[83] Tripathi P., Ansari M.A., Gandhi T.K., Mehrotra R., Bin Heyat B., Akhtar F., Ukwuoma C.C., Muaad A.Y., Kadah Y.M., Al-Antari M.A. (2022). Ensemble Computational Intelligent for Insomnia Sleep Stage Detection via the Sleep ECG Signal. IEEE Access.

